# Identification of sugar transporter genes and their roles in the pathogenicity of *Verticillium dahliae* on cotton

**DOI:** 10.3389/fpls.2023.1123523

**Published:** 2023-01-26

**Authors:** Lihua Chen, Bin Chen, Qian-Hao Zhu, Xinyu Zhang, Tiange Sun, Feng Liu, Yonglin Yang, Jie Sun, Yanjun Li

**Affiliations:** ^1^ The Key Laboratory of Oasis Eco-agriculture, Agriculture College, Shihezi University, Shihezi, Xinjiang, China; ^2^ CSIRO Agriculture and Food, Canberra, Australia; ^3^ Cotton Research Institute, Shihezi Academy of Agricultural Sciences, Shihezi, China

**Keywords:** cotton, verticillium dahliae, sugar transporter, HIGS, asiRNA

## Abstract

**Introduction:**

Verticillium wilt (VW) caused by *Verticillium dahliae* is a soil-borne vascular fungal disease that severely affects cotton yield and fiber quality. Sugar metabolism plays an important role in the growth and pathogenicity of *V. dahliae*. However, limited information is known about the sugar transporter genes and their roles in the growth and pathogenicity of *V. dahliae*.

**Method:**

In this study, genome-wide identification of sugar transporter genes in *V. dahliae* was conducted and the expression profiles of these genes in response to root exudates from cotton varieties susceptible or resistant to *V. dahliae* were investigated based on RNA-seq data. Tobacco Rattle Virus-based host-induced gene silencing (TRV-based HIGS) and artificial small interfering RNAs (asiRNAs) were applied to investigate the function of candidate genes involved in the growth and pathogenic process of *V. dahliae*.

**Results:**

A total of 65 putative sugar transporter genes were identified and clustered into 8 Clades. Of the 65 sugar transporter genes, 9 were found to be induced only by root exudates from the susceptible variety, including *VdST3* and *VdST12* that were selected for further functional study. Silencing of *VdST3* or *VdST12* in host plants by TRV-based HIGS reduced fungal biomass and enhanced cotton resistance against *V. dahliae*. Additionally, silencing of *VdST12* and *VdST3* by feeding asiRNAs targeting *VdST12* (asiR815 or asiR1436) and *VdST3* (asiR201 or asiR1238) inhibited fungal growth, exhibiting significant reduction in hyphae and colony diameter, with a more significant effect observed for the asiRNAs targeting *VdST12*. The inhibitory effect of asiRNAs on the growth of *V. dahliae* was enhanced with the increasing concentration of asiRNAs. Silencing of *VdST12* by feeding asiR815+asiR1436 significantly decreased the pathogenicity of *V. dahliae*.

**Discussion:**

The results suggest that *VdST3* and *VdST12* are sugar transporter genes required for growth and pathogenicity of *V. dahliae* and that asiRNA is a valuable tool for functional characterization of *V. dahliae* genes.

## Introduction

Cotton is one of the most important economic crops in the world, the main source of natural fiber for the textile industry, and an important strategic material for the national livelihood (Zhang et al., 2015). The cotton quality and yield are often affected by various biotic and abiotic stresses. Cotton Verticillium Wilt (VW), caused by *Verticillium dahliae* Kleb., is one of the most serious cotton diseases worldwide (Fradin and Thomma, 2006). This disease can result in more than 50% cotton fields damaged and cause substantial economic loss every year in China (Zhang et al., 2020). *V. dahliae* is particularly difficult to control because it persists in soil as long-living dormant microsclerotia (Fradin and Thomma, 2006; Luo et al., 2014). *V. dahliae* invades cotton through the root system (Duressa et al., 2013). After sensing cotton root exudates, the microconidia of *V. dahliae* germinate towards roots and then produce hyphae, which enter the root epidermal cells and multiply in the xylem vessels. Mycelium, spores, or polysiccharedes produced by *V. dahliae* can clog the vessels, resulting in leaf yellowing, wilt, necrosis, defoliation and vascular brown coloration (Song et al., 2020). In recent years, with the completion of whole genome sequencing of *V. dahliae*, a number of genes involved in the growth and pathogenic process of *V. dahliae* have been identified (Gui et al., 2017; Zhang et al., 2017; Qin et al., 2018; Xu et al., 2018; Zhang et al., 2018). However, due to the complexity of the molecular basis of pathogenicity in *V. dahliae*, we expect more genes involved in the pathogenic process of *V. dahliae* to be found.

During the infection process, pathogenic fungi need to use various metabolites secreted by the host to provide nutrients and energy. Sugar is an essential nutrient and a major component for living organisms. Sugar metabolism plays an important role in the growth and pathogenicity of *V. dahliae*. During the last decades, cell wall degrading enzymes which degrade cell wall polysaccharides (cellulose, hemicellulose and pectin) have been extensively studied (Fradin and Thomma, 2006). The cell wall degrading enzyme genes, such as *VdEg-1*, *VdSSP1* and *VdSNF1*, have been proved to be related to the pathogenicity of *V. dahliae* (Novo et al., 2006; Maruthachalam et al., 2011; Tzima et al., 2011; Liu et al., 2013a). The sugar transmembrane transportation is mainly carried out by sugar transporters, which are responsible for taking up monosaccharides and short oligosaccharides derived from plant cell wall polysaccharides (Doidy et al., 2012; Peng et al., 2018). However, the sugar transporter (ST) genes have not yet been investigated in *V. dahliae*.

Sugar transporters widely exist in all kingdoms of life from microorganisms to plants and animals. Sugar transporters belong to the major facilitator superfamily (MFS), usually composed of 400 to 600 amino acids. They are highly similar in primary structure and usually contain 12 transmembrane domains (Law et al., 2008). Sugar transporters mediate the transport of monosaccharides (such as glucose, frucotse and mannose), sucrose and polyols (such as mannitol and sorbitol) (Mahmud and Kissinger, 2017). Monosaccharide transporters can be clustered into hexose, pentose, and inositol based on a phylogenetic relationship analysis (Peng et al., 2018). Many sugar transporter genes have been identified from different fungus (Saitoh et al., 2014; Schuler et al., 2015). *ST* genes have been found to participate in the interactions between host plants and fungus and to perform an important function in the absorption of host sugars (Doehlemann et al., 2005; Fang and St Leger, 2010; Doidy et al., 2012). Knockout or silencing of sugar transporter genes identified in some fungi affected the growth and development of the fungi and reduced their pathogenicity (Wahl et al., 2010; Liu et al., 2013b; Saitoh et al., 2014; Chang et al., 2020).

It has been found that the content of glucose, fructose and sucrose in the root exudates from cotton varieties susceptible to *V. dahliae* is much higher than that from resistant ones (Wu et al., 2007). Previous transcriptome analysis found that several *ST* genes responded to root exudates from susceptible cotton variety, suggesting that they were closely related to the pathogenicity of *V. dahliae* (Zhang et al., 2020). In order to explore the role of *ST* genes in growth and pathogenicity of *V. dahliae*, here, genome-wide identification of *ST* genes was conducted and their expression profiles after sensing root exudates from cotton varieties susceptible or resistant to *V. dahliae* were analyzed. A total of 65 *VdST* genes were identified and 9 of them were found to be induced by root exudates from susceptible cotton variety. Two *VdST* genes (*VdST3* and *VdST12*) were selected for functional study by using host-induced gene silencing (HIGS) and asiRNA (artificial small interfering RNA) technologies. The results indicated that silencing *VdST3* or *VdST12* resulted in a reduced pathogenicity of *V. dahliae* and increased cotton resistance to VW, demonstrating the importance of the two genes in pathogenicity of *V. dahliae*.

## Materials and methods

### Fungi and plant materials and growth conditions

The strongly pathogenic strain Vd991 of *V. dahliae* was used in this study. The Vd991 strain was cultured in 200 mL of Czapek liquid media and incubated for 5-7 d at 25°C with 150 rpm/min shaking. The spores were collected by filtering the fungal solution with sterilized gauze (8 layers) and were adjusted to 1.0×10^7^ CFU/mL or 1.0×10^5^ CFU/mL using a hemocytometer.

The Upland cotton variety Xinluzao 7 susceptible to *V. dahliae* was used in this study. Cotton seeds were grown in pots and placed in a controlled environmental chamber under a photoperiod of 16h of light and 8h of darkness at 28°C. Seedlings at the second true leaf stage were used for infection assays, in which the growth temperature was changed to 25°C for better development of disease symptoms.

### RNA extraction and cDNA synthesis

Total RNA of *V. dahliae* was extracted using Fungal RNA Kit (Omega Inc., USA) according to the manufacturer’s procedures. Total RNA of cotton tissues was extracted using the EASYspinPlus Plant RNA Extraction Kit (Aidlab, Beijing, China). Easyscript^®^ One-step gDNA Removal and cDNA Synthesis Super Mix (TransGen Biotech, Beijing, China) kit was used to synthesize cDNA.

### Identification of the sugar transporter family genes of *V. dahliae*

The gtf, genomic, CDS, and protein sequences of *V. dahliae* (ASM15067v2) were downloaded from the *V. dahliae* data website (https://fungi.ensembl.org/Verticillium_dahliae/Info/Index). The Sugar_tr domain (PF00083) downloaded from Pfam database (https://www.ebi.ac.uk/interpro/entry/pfam/#table) was used to search the sugar transporter proteins in *V. dahliae* protein database by HMMER software with a standard hmmsearch score ≥ 238 (Peng et al., 2018). Gene ID and chromosome location of *VdST* genes were obtained from the NCBI database (https://www.ncbi.nlm.nih.gov/). The Sequence Manipulation Suite online tool (http://www.detaibio.com/sms2/protein_iep.html) was used to estimate the basic physicochemical properties of the VdST proteins, such as protein length (PL), molecular weight (MW) and isoelectric point (pI). Transmembrane structural domains (TMD) were predicted with TMHMM program (http://genome.cbs.dtu.dk/services/tmhmm) and the subcellular localization of VdST proteins was predicted using the online software Prot Comp 9.0 (http://www.softberry.com/berry.phtml?topic=protcompan&group=programs&subgroup=proloc).

### Multiple sequence alignment and phylogenetic tree analyses

The ST protein sequences from *V. dahliae* and other fungi (**Table S1**) were initially aligned using Clustal W. The phylogenetic analysis was accomplished using MEGA 7.0 *via* the neighbor-joining (NJ) method and bootstrap tests replicated by 1000 times. Finally, the tree was visualized by the Interactive Tree Of Life online tool (https://itol.embl.de/).

### Analyses of the conserved motifs and structure of *VdST* genes

The conserved motifs of the VdST proteins were analyzed by the MEME program (https://meme-suite.org/meme/tools/meme) using the parameters of 10 motifs and displayed by TBtools software (Chen et al., 2020). The prepared gtf format file and gene sequence number were put into TBtools for gene structure visualization.

### Analysis of the expression profile of *VdST* genes based on RNA-seq datasets

Previous RNA-seq datasets (BioProject accession ID: PRJNA545805) were used to explore the expression profiles (FPKM, fragments per kilobase per million fragments mapped) of *VdST* genes. The RNA-seq datasets were generated from *V. dahliae* samples cultured on root exudates from an Upland cotton variety Xinluzao 7 (X) susceptible to *V. dahliae*, a Sea Island cotton variety Hai7124 (H) resistant to *V. dahliae*, or water (W) for 0h, 6h, 12h, 24h and 48h (Zhang et al., 2020). The gene expression heatmap of *VdST* genes was drawn using TBtools software.

### Host-induced silencing of *VdST* genes

The pTRV1, pTRV2 and pTRV2-*GhCHLI* plasmids were kindly provided by Prof. Longfu Zhu of Huazhong Agricultural University. Four interfering fragments *VdST3-*1 (337bp), *VdST3-*2 (346bp), *VdST12*-1 (346bp) and *VdST12*-2 (367bp) designed to target *VdST3* (Gene ID: VDAG_07563) or *VdST12* (Gene ID: VDAG_04513) were amplified from Vd991 cDNA with *VdST3*-F1/R1, *VdST3*-F2/R2, *VdST12*-F1/R1 and *VdST12*-F2/R2 (**Table S2**) and inserted into pTRV2 vector, respectively. The HIGS (host-induced gene silencing) vectors (pTRV2-*VdST3-*1, pTRV2-*VdST3-*2, pTRV2-*VdST12*-1 and pTRV2-*VdST12*-2) were generated and transformed into *Agrobacterium tumefaciens* strain GV3101 by electroporation. Cotton leaves (Xinluzao 7) were used for injection with TRV as previous description (Xiong et al., 2020). The pTRV2-*GhCHLI* treated seedlings were applied as a positive control. When the bleaching phenotype was observed in pTRV2-*GhCHLI* treated seedlings, the pTRV2-*VdST3-*1, pTRV2-*VdST3-*2, pTRV2-*VdST12*-1 and pTRV2-*VdST12*-2 treated plants were inoculated with Vd991 by root irrigation with 20 mL spore suspension (1×10^7^ CFU/mL). The fungal infection symptoms were investigated at 14 and 21 dpi (days post inoculation). The disease index (DI) was calculated according to a five-scale classification (0, 1, 2, 3 and 4) of VW disease on cotton seedlings (Standard No.: GB/T28084-2011).

### asiRNA design and treatment

Multiple online sites (http://biodev.extra.cea.fr/DSIR/DSIR.html, https://www.invivogen.com/sirnawizard/design.php) were used for siRNA design. Sequences in two different sites specific to each gene were used as the asiRNA candidates. The specificity of the asiRNA sequences were confirmed by BLASTn against the genomic sequences of *V. dahliae* to avoid off-targeting. A 19-bp sequence specific to the nematode genome was used as negative control (NC). Double T nucleotides were added to the 3’-terminus of these candidate sequences to stabilize the asiRNAs (**Table S3**). The double-stranded asiRNA sequences were synthesized by Shanghai Sangon Biotech (China).

The Vd991 strain was incubated in Czapek liquid media containing asiRNA at different concentrations (0, 50, 100 or 200 nM) for 6 d at 25°C with 150 rpm/min shaking. The spores co-cultured with asiRNA were collected and adjusted to 1×10^7^ CFU/mL. Then 10 µL of spore suspension containing asiRNA was inoculated into the center of PDA (Potato Dextrose Agar) medium and incubated at 25°C in the dark. The colony diameter was measured at 3, 6, 9, 12, 15 and 18 days post incubation. To observe hyphal morphology, 10 µL of spore suspension (1×10^5^ CFU/mL) containing asiRNA at different concentrations was applied to PDA medium. The hyphal morphology was observed at 36 hours post incubation under microscope. Wild-type Vd991 was used as control (CK), and Vd991 co-cultured with asiRNA from nematode was used as a negative control (NC). All tests were repeated three times.

To investigate the role of *VdST3* and *VdST12* in carbon utilization, 6 different monosaccharides, disaccharides or polysaccharides, including glucose (50 g/L), galactose (50 g/L), xylose (50 g/L), maltose (50 g/L), sucrose (50 g/L) and cellulose (10 g/L) were individually added to Czapek Dox medium lacking carbon source. Then, 10 µL of spore suspension (1×10^7^ CFU/mL) from Vd991 co-cultured with asiRNA (200 nM) was placed in media with different carbon sources, and then incubated at 25°C in the dark. The colony diameter was measured at 3, 6, 9, 12, and 15 days post incubation. All tests were repeated three times.

To investigate whether asiRNAs affect the pathogenicity of *V. dahliae*, spore suspension (1×10^7^ CFU/mL) from Vd991 co-cultured with asiRNAs (200 nM) was prepared for infection process assay. Wild-type Vd991 was used as control (CK), and Vd991 co-cultured with asiRNA from nematode was used as a negative control (NC). Xinluzao 7 seedlings at two-leaf stage were inoculated with various Vd991 by root irrigation with 20 mL spore suspension. The fungal infection symptoms were investigated at 14 and 21dpi. The disease index (DI) was calculated as mentioned above.

### Gene expression assay

Roots, stems and leaves from infected seedlings were sampled at 14 and 21dpi for RNA extraction. The Vd991 strain incubated in Czapek liquid media containing asiRNA at different concentrations for 6 d were collected for RNA extraction. The transcription levels of *VdST3* and *VdST12* were analyzed by qRT-PCR with primer pair of *VdST3*-qF1/R1 and *VdST12*-qF1/R1 (**Table S2**), respectively. Cotton *Tubulin* gene was used as internal reference. The qRT-PCR assay was conducted using the SYBR Green Mix (TaKaRa, Dalian, China), and PCR cycling started with an initial step of 95°C for 10s, 40 cycles at 60°C for 15s, and 72°C for 20s. The qRT-PCR reactions were performed on a Roche LightCycler 480 II instrument and the results were analyzed by the 2^-ΔΔCT^ method (Livak and Schmittgen, 2001). The primer specificity and the formation of primer-dimers were tested by dissociation curve analysis.

### Recovery of *V. dahliae* from infected seedlings

At 14 dpi, 10 infected seedlings were randomly selected for the *V. dahliae* recovery experiment. Stems were harvested by cutting the seedlings from the base and cut into 2 cm long segments. The stem segments were sterilized with 75% alcohol for 30 s, then soaked in 0.1% HgCl_2_ solution for 5 min and rinsed 3-5 times in sterile water. The sterilized stem segments were evenly placed on PDA plates and incubated at 25°C for colony observation at 7 days post incubation.

### Fungal biomass measurement

At 21 dpi, different tissues from infected seedlings were collected separately and used for measurement of fungal biomass by qRT-PCR. DNA was extracted from roots, stems and leaves by CTAB method. *V. dahliae* specific primers ITS1-F and ST-Ve1-R were used for fungal biomass measurement as previously reported (Xiong et al., 2020). To normalize differences in DNA template amounts, the cotton *GhUBQ7* gene (DQ116441.1) amplified using primer pair UBQ7-F/R was used as the internal reference. qRT-RCR reactions were performed as described above.

## Results

### Genome-wide identification of *ST* genes in *V. dahliae*

A total of 65 putative *ST* genes were identified in the genome of *V. dahliae* by HMMER analysis and named *VdST1* to *VdST65* (**Table S4**). The length of these VdST protein sequences ranged from 396 to 669 amino acids (aa), with the predicted molecular weights (MW) from 42.94 to 73.43 kDa, theoretical isoelectric points (*p*I) from 5.29 to 9.53, and the number of transmembrane domains (TMD) ranged from 7 to 12. It was found that 29 out of the 65 VdST proteins contained the entire 12 TMDs, 28 possessed 10 or 11 TMDs, while 8 carried only 7 to 9 TMDs. The 65 *VdST* genes were randomly distributed on 8 chromosomes, of which chromosomes 3 and 4 harbored the most *VdST* genes (12 and 10, respectively), whereas chromosomes 8 and 7 contained only 3 and 5 *VdST* genes, respectively. According to the subcellular localization predictions, overwhelming majority of VdST proteins (60) were located in the plasma membrane, with a few localized to endoplasmic reticulum, vacuole, mitochondrion, golgi and nucleus.

### Classification and phylogenetic analysis of *VdST* genes

**Figure 1:**
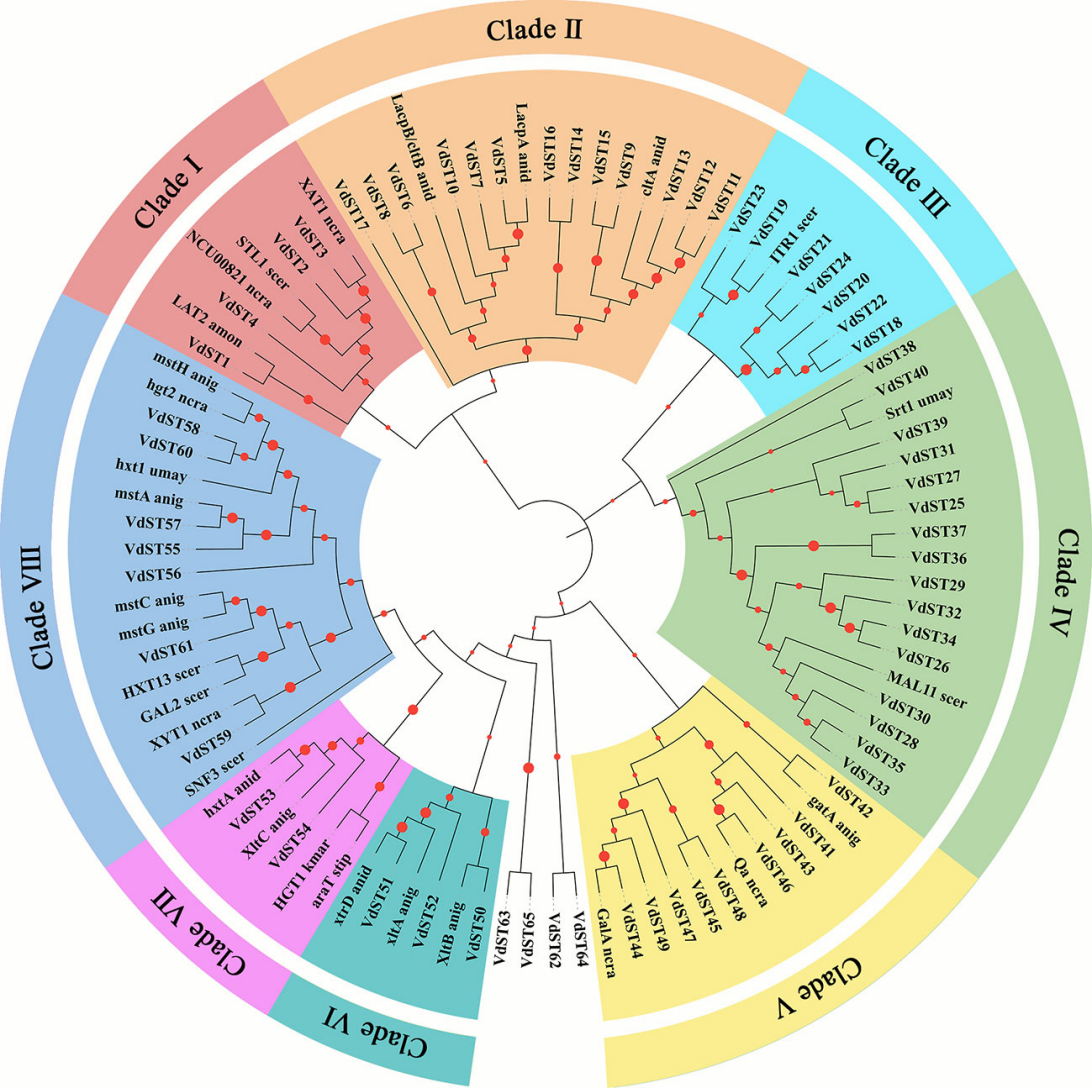
Phylogenetic classification of sugar transporters in *V. dahliae*. The phylogenetic tree contained 65 VdST proteins and 30 ST proteins from other fungi (**Table S1**). The tree was generated by MEGA 7.0 with 1000 bootstrap replications. Eight Clades were distinguished by different colors. The abbreviation of fungal species name is attached to each transporter protein (anid = *Aspergillus nidulans*, anig = *Aspergillus niger*, amon = *Ambrosiozyma monospora*, bcin = *Botrytis cinerea*, kmar = *Kluyveromyces marxianus*, ncra = *Neurospora crassa*, scer = *Saccharomyces cerevisiae*, spas = *Saccharomyces pastorianus*, stip = *Scheffersomyces stipitis*, umay = *Ustilago maydis*).

The protein sequences of all 65 *VdST* genes together with 30 *ST* genes reported in other fungi (**Table S1**) were used for phylogenetic analysis (Peng et al., 2018). As shown in **Figure 1**, 65 *VdST* genes were classified into 8 different Clades. There were 16 *VdST* genes in Clade IV, which was the largest subfamily, including sucrose transporter *Srt1* from *Ustilago maydis* and maltose transporter *MAL11* from yeast (*Saccharomyces cerevisiae*). Clade II contained 13 *VdST* genes that were clustered with known lactose permease and hexose transporter genes, such as lactose transporter *LacpA*, *LacpB*/*cltB* and cellobiose transporter *cltA* from *Aspergillus nidulans*. Clade V included 9 *VdST* genes and quinate permease-encoding genes from other species, including D-galacturonic acid transporters *GalA* (*Neurospora crassa*) and *gatA* (*Aspergillus niger*) and quinic acid transporter *Qa* (*Neurospora crassa*). Clade VIII included 7 *VdST* genes and glucose transporter genes from other fungi, such as hexose transporter *HXT13* (*Saccharomyces cerevisiae*) and *hxt1* (*Ustilago maydis*), glucose transporter *hgt2* (*Neurospora crassa*), *SNF3* (*Saccharomyces cerevisiae*), *mstC*, *mstG*, *mstA* and *mstA* (*Aspergillus niger*), galactose transporter *GAL2* (*Saccharomyces cerevisiae*) and pentose transporter *XYT1* (*Neurospora crassa*). Clade VIII harbored 7 *VdST* genes and myoinositol transporter *ITR1* from *Saccharomyces cerevisiae*. Other subfamilies, including Clade I, VI and VII, contained only 2 to 4 *VdST* genes. Four genes (*VdST62*, *VdST63*, *VdST64* and *VdST65*) were not classified into any Clade.

### Conserved motifs and structure of *VdST* genes

**Figure 2:**
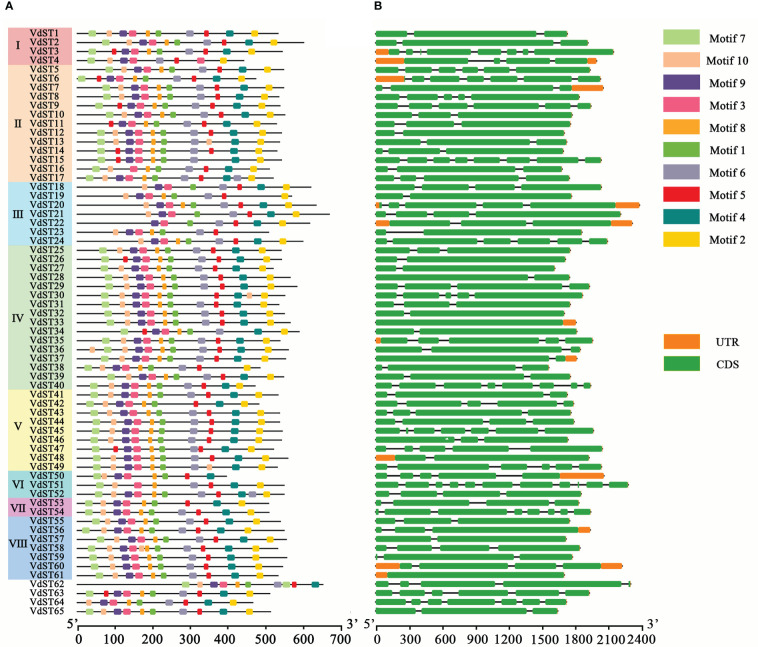
Conserved motifs and exon-intron structure of *ST* genes in *V. dahliae*. **(A)** Conserved motifs of the VdST proteins were identified by MEME program. Protein sequences and conserved motifs were represented by black lines and differently colored boxes, respectively. **(B)** Exon-intron structure of the 65 *ST* genes identified in *V. dahliae*. Untranslated regions, exons and introns were indicated by orange boxes, green boxes and black lines, respectively.

A total of 10 conserved motifs were identified in VdST proteins, and the location of these motifs in each protein was showed in **Figure 2A**. The motif numbers varied from 7 (VdST22 and VdST23) to 11 (VdST17, VdST36, VdST52 and VdST58), and most proteins (44) harbored 10 motifs. Compared with the VdST proteins in other Clade, the VdST proteins in Clade III contained less motifs, ranging from 7 to 9. Motif 3 was identified in all 65 VdST proteins, suggesting that it may be critical for the role of VdST proteins. Most proteins (more than 61) contained motifs 1, 2, 4, 5, 6, 8 and 9 (**Figure 2A** and **Table S5**), suggesting their importance for the function of VdST proteins. Motif 7 was absent in all proteins of Clade III, and motif 10 was absent in several Clade III VdST proteins. Interestingly, where there is a deficiency in motif 5 it is usually replaced by motif 10, and vice versa, lack of motif 10 is usually replaced by motif 5, such as in VdST3 and VdST13, suggesting that these two motifs may be structurally and functionally similar, complementing each other. The length of the 10 conserved motifs ranged from 13 to 21 amino acids, and the putative Sugar_tr structural domain was predicted in the conserved motifs 1-7 and 9 (**Table S5**). The amino acid frequency of the 10 motifs was not consistent in different VdST proteins (**Figure S1**).

To better understand the structure of *VdST* genes, their exons and introns were analyzed (**Figure 2B**). There was no obvious similarity in the arrangement and number of exons and introns in each Clade. The number of exons (1 to 11) and introns (0 to 10) in 65 *VdST* genes were found to be variable. Most *VdST* genes (51) contained 2 to 5 exons, 12 genes contained more than 6 exons, and 2 genes contained only 1 exon. Additionally, the length of exons was also found to be variable, whereas the length of introns was shorter and more conserved.

### Responses of *VdST* genes to root exudates from cotton varieties susceptible or resistant to *V. dahliae*

**Figure 3:**
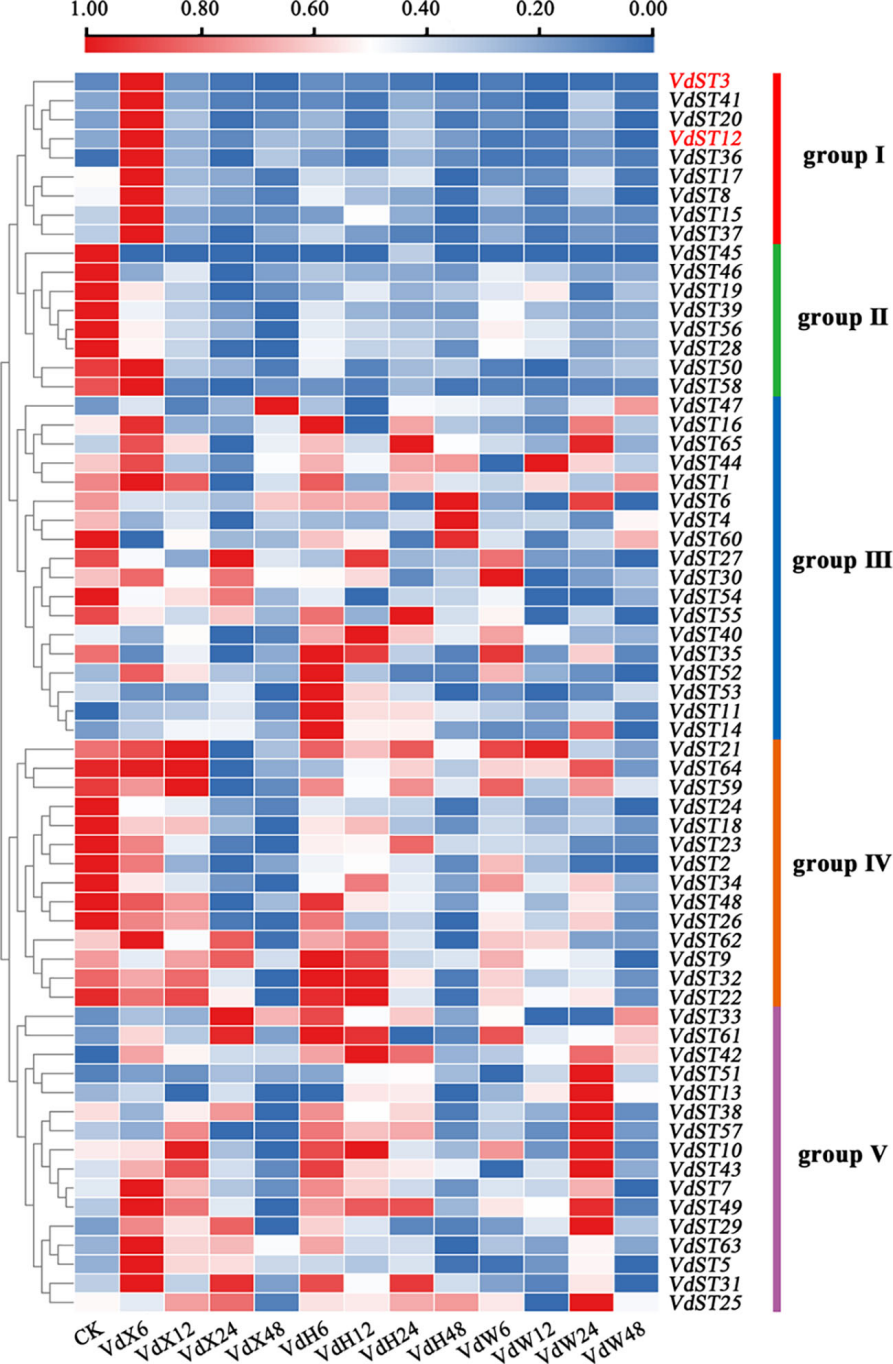
Expression profile analysis of the 65 *VdST* genes after sensing root exudates from different cotton varieties. VdX6, 12, 24, and 48 represented *V. dahliae* samples cultured by root exudates from the susceptible cotton variety (Xinluzao 7) for 6, 12, 24 and 48h, respectively. VdH6, 12, 24, and 48 represented *V. dahliae* samples cultured by root exudates from the resistant cotton variety (Hai7124) for 6, 12, 24 and 48h, respectively. VdW6, 12, 24, and 48 represented *V. dahliae* samples cultured in water for 6, 12, 24 and 48h, respectively. The color of the scale bar, ranging from blue to red, represented low to high expression. The two genes (*VdST3* and *VdST12*) selected for further study were highlighted in red.

To find the *VdST* genes involved in pathogenic process of *V. dahliae*, the expression profiles of the 65 *VdST* genes in response to root exudates from two varieties (a susceptible Upland cotton variety Xinluzao 7 and a resistant Sea Island cotton variety Hai7124) were investigated by using the RNA-seq datasets available from our previous research (Zhang et al., 2020). Finally, a heatmap was generated based on FPKM value of the 65 *VdST* genes, exhibiting the expression profiles of these genes after sensing root exudates from different varieties. As shown in **Figure 3**, the *VdST* genes could be divided into 5 groups based on their expression profiles. It was notable that group I contained 9 *VdST* genes (*VdST3*, *VdST41*, *VdST20*, *VdST12*, *VdST36*, *VdST17*, *VdST8*, *VdST15* and *VdST37*), which exhibited high expression at 6 hours after sensing root exudates from susceptible cotton variety (VdX6) but had no response to root exudates from resistant cotton variety (VdH), suggesting that these *VdST* genes may play important roles in the pathogenicity of *V. dahliae*. To verify this speculation, two *VdST* genes, *VdST3* (VDAG_07563) and *VdST12* (VDAG_04513), were selected for further characterization.

### Host-induced silencing of *VdST3* or *VdST12* alleviates disease symptoms caused by *V. dahliae* infection

TRV-based host-induced gene silencing (HIGS) was adopted to silence *VdST3* or *VdST12* genes in *V. dahliae*. Two interfering fragments were designed for each gene to silence *VdST3* (*VdST3*-1 and *VdST3*-2) or *VdST12* (*VdST12*-1 and *VdST12*-2). Ten days after injection with the HIGS vectors, the seedlings (Xinluzao 7) injected with pTRV2-*GhCHLI* showed the leaf-bleaching phenotype in the newly emerging leaves (**Figure S2**), indicating that the TRV-based technique worked well.

**Figure 4:**
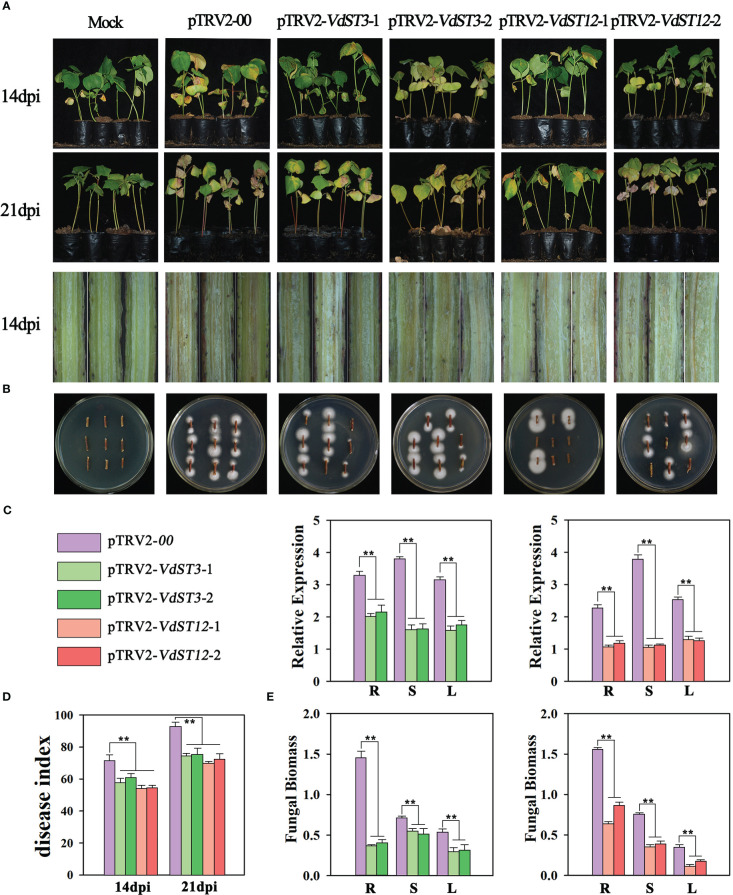
Functional assessment of *VdST3* and *VdST12* in the pathogenicity of *V. dahliae* by TRV-based HIGS. **(A)** Fungal infection symptoms of HIGS treated seedlings (pTRV2-*VdST3-*1, pTRV2-*VdST3-*2, pTRV2-*VdST12*-1 and pTRV2-*VdST12*-2) at 14 and 21 dpi. **(B)** Fungal recovery from the stem segments of HIGS treated seedlings. Stem segments were harvested at 14 dpi, plated on PDA medium and incubated at 25°C. Photos were taken at 7 days post incubation. **(C)** The expression level of *VdST3* and *VdST12* in HIGS treated seedlings at 21 dpi by qRT-PCR analysis. Total RNA was isolated from roots (R), stems (S) and leaves (L) of HIGS treated seedlings at 21 dpi. The cotton *tubulin* gene was used as the internal reference. **(D)** Disease index of HIGS treated seedlings at 14 and 21 dpi. **(E)** qRT-PCR measurement of fungal biomass in HIGS treated seedlings at 21 dpi. The data were statistically analyzed by the IBM SPSS statistics 26.0. Statistical significance was determined using Student’s t-test. Asterisks (**) above the error bars indicated significant difference at p < 0.01 between HIGS treated seedlings and control (pTRV2-00 treated seedlings).

When cotton seedlings injected with pTRV2-*GhCHL1* displayed leaf-bleaching phenotype, the seedlings injected with HIGS vector were inoculated with Vd991 by the root irrigation method. Fungal infection symptoms were investigated at 14 and 21 dpi (days post inoculation). At 14 dpi, pTRV2-00 seedlings (control) showed obvious leaf yellowing and wilting phenotype, however, the HIGS treated seedlings displayed only mild leaf yellowing phenotype (**Figure 4A**). The disease index (DI) of pTRV2-*VdST3-*1 (DI=55.3), pTRV2-*VdST3-*2 (DI=61.6), pTRV2-*VdST12*-1 (DI=51.5) and pTRV2-*VdST12*-2 (DI=52.2) seedlings was significantly lower than that of pTRV2-00 seedlings (DI=68.2) (**Figure 4D**). At 21 dpi, pTRV2-00 seedlings showed severe defoliation symptom, while the HIGS treated seedlings displayed only obvious leaf yellowing and wilting but few defoliation symptoms (**Figure 4A**). The disease index of pTRV2-*VdST3-*1 (DI=70.5), pTRV2-*VdST3-*2 (DI=72.3), pTRV2-*VdST12*-1 (DI=66.4) and pTRV2-*VdST12*-2 (DI=68.9) seedlings was significantly lower than that of control plants (DI=88.1) (**Figure 4D**). Stem dissection revealed that the HIGS treated seedlings had significantly lighter browning than pTRV2-00 seedlings (**Figure 4A**).

To test the silencing efficiency of TRV-based HIGS, qRT-PCR was used to determine the relative expression level of *VdST3* and *VdST12* at 21 dpi. Compared with pTRV2-00 seedlings, the expression level of *VdST3* in pTRV2-*VdST3-*1 and pTRV2-*VdST3-*2 seedlings, and that of *VdST12* in pTRV2-*VdST12*-1 and pTRV2-*VdST12*-2 seedlings were reduced significantly in all tissues (root, stem and leaf) (**Figure 4C**), indicating that TRV-based HIGS worked well to silence *V. dahliae* genes in infected cotton seedlings.

*V. dahliae* was isolated from pTRV2-*VdST3-*1, pTRV2-*VdST3-*2, pTRV2-*VdST12*-1 and pTRV2-*VdST12*-2 seedlings at 14 dpi, and colony growth was observed at 7 days after incubation on PDA medium. The average spread size of colony grown from stems of all seedlings injected with HIGS vector was reduced compared to that from stems of pTRV2-00 seedlings (**Figure 4B**). At 21 dpi, total DNA were extracted from roots, stems and leaves of the HIGS treated seedlings for measurement of fungal biomass using qRT-PCR. Fungal biomass quantifications revealed that less fungal biomass accumulated in pTRV2-*VdST3-*1, pTRV2-*VdST3-*2, pTRV2-*VdST12*-1 and pTRV2-*VdST12*-2 seedlings than in the pTRV2-00 seedlings (**Figure 4E**). Taken together, down-regulation of *VdST3* and *VdST12* by TRV-based HIGS significantly inhibited accumulation of fungal biomass in cotton seedlings and enhanced cotton resistance against *V. dahliae*, suggesting that *VdST3* and *VdST12* are involved in pathogenic process of *V. dahliae*.

### Growth of *V. dahliae* is inhibited by application of asiRNAs targeting *VdST3* or *VdST12*

**Figure 5:**
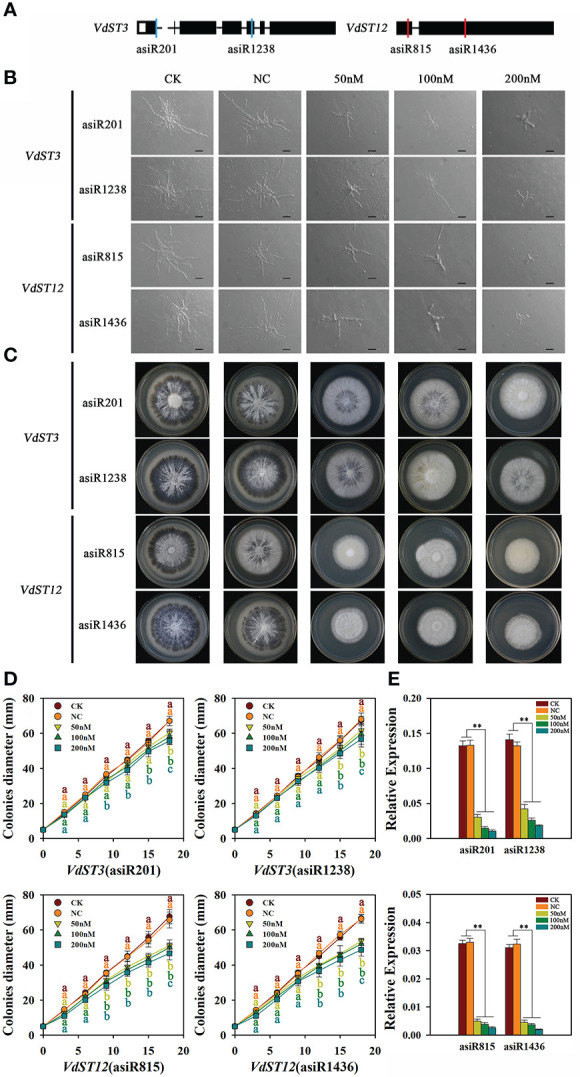
Effect of asiRNAs targeting *VdST3* and *VdST12* on fungal hyphae and colony morphology. **(A)** The target sites of asiRNAs in *VdST3* and *VdST12*. Black boxes indicated exons, and red and blue lines represented the target sits. **(B)** Effect of asiRNAs targeting *VdST3* or *VdST12* on hyphal growth. The Vd991 strain was incubated in Czapek liquid medium containing asiRNA at different concentrations (0, 50, 100 or 200 nM) before inoculating on PDA medium. Wild-type Vd991 was used as control (CK), and Vd991 co-cultured with nematode asiRNA was used as negative control (NC). The images were taken after 36 hours of spore incubation on PDA medium. Bares=50μm. **(C)** Effect of asiRNAs targeting *VdST12* and *VdST3* on colony morphology. The images were taken at 18 days post incubation on PDA medium. **(D)** Effect of asiRNAs targeting *VdST3* or *VdST12* on growth rate of fungal colony. **(E)** The expression level of *VdST3* and *VdST12* in Vd991 co-cultured with different concentrations of asiRNAs by qRT-PCR analysis. The Vd991 strain incubated in Czapek liquid medium containing asiRNA at different concentrations for 6 d were collected for RNA extraction. Values were means ± SD from three replicates. The above results were obtained in at least three independent experiments. The data were statistically analyzed by the IBM SPSS statistics 26.0. Significant difference in different treatments was analyzed using Duncan’s multiple range tests (different letters above the error bars indicated statistically different at p<0.05) for one way ANOVA. An asterisk (**) above the error bars indicates that there is a significant difference in gene expression between the strains treated with different concentrations of asiRNA and CK and NC (p<0.01).

In order to test whether the growth of *V. dahliae* could be inhibited by *in vitro* treatment with asiRNAs that target *VdST3* and *VdST12*, the hyphae and colony morphology of Vd991 co-cultured with different concentrations of asiRNAs were observed. Compared with Vd991 without asiRNA (CK) or co-cultured with nematode asiRNA (NC), the Vd991 co-cultured with asiRNA (asiR815 or asiR1436) targeting *VdST12* (**Figure 5A**) showed an obvious reduction of fungal hyphae and a significantly slow growth of colonies (**Figure 5B**). At 18 days post incubation, compared with the CK, the colony diameter of Vd991 co-cultured with asiR815 at the concentration of 50, 100 and 200 nM reduced by 24.6%, 26.1% and 31%, respectively. For asiR1436, the corresponding reduction rate was 19.5%, 22.0% and 28.8%, respectively (**Figure 5C, D**). These results suggest that the asiRNAs targeting *VdST12* effectively inhibited the growth of *V. dahliae*. The asiRNAs (asiR201 or asiR1238) targeting *VdST3* (**Figure 5A**) could inhibit fungal hyphae and colony growth but with a less inhibitory effect compared to the asiRNAs targeting *VdST12* (**Figures 5B, C**). At 18 days post incubation, compared with the CK, the colony diameter of the asiR201 treatment reduced by 10.0%, 13.4% and 17.0%, and the asiR1238 treatment by 11.4%, 12.9% and 15.8% at the concentration of 50, 100 and 200 nM, respectively (**Figure 5C, D**). But in both cases, the inhibitory effect of asiRNAs on *V. dahliae* was positively correlated with the concentration of asiRNAs. The qRT-PCR results showed that the expression level of *VdST3* and *VdST12* in Vd991 co-cultured with different concentrations of asiRNAs was significantly lower than that of CK and NC (**Figure 5E**), suggesting that asiRNAs could effectively inhibit gene expression in *V. dahliae*.

### Carbon utilization of *VdST3* and *VdST12*

**Figure 6:**
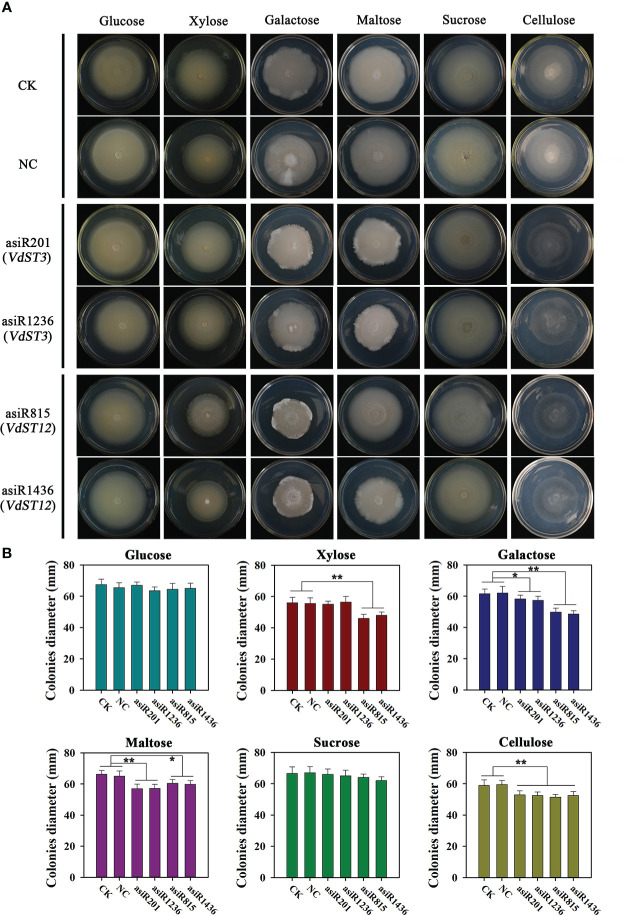
Morphology and diameter of colony from asiRNAs-treated strain on Czapek Dox medium with different carbon sources. **(A)** Colony morphology on Czapek Dox medium with different carbon sources. The Vd991 was co-cultured with asiRNA (200 nM) before inoculating on Czapek Dox medium. Wild-type Vd991 was used as control (CK), and Vd991 co-cultured with nematode asiRNA was used as negative control (NC). The images were taken at 15 days post incubation on PDA medium. **(B)** Colony diameter on Czapek Dox medium with different carbon sources. Values were means ± SD from three replicates. The above results were obtained in at least three independent experiments. The data were statistically analyzed by the IBM SPSS statistics 26.0. Statistical significance was determined using Student’s t-test. Asterisks (* and **) above the error bars indicated significant difference at p < 0.05 and p < 0.01 between asiRNA-treated strain and CK and NC.

To investigate the role of *VdST3* and *VdST12* in carbon utilization, Vd991 co-cultured with asiRNAs (200 nM) was incubated separately in Czapek Dox medium containing different carbon sources. As shown in **Figures 6A, B**, Vd991 co-cultured with asiRNAs targeting *VdST12* (asiR815 or asiR1436) showed a reduced colony growth on medium containing xylose, galactose, maltose and cellulose, but was not affected on medium containing glucose and sucrose, suggesting that *VdST12* was involved in the utilization of xylose, galactose, maltose and cellulose. The growth of *V. dahliae* co-cultured with asiRNAs targeting *VdST3* (asiR201 or asiR1238) was reduced on medium containing galactose, maltose and cellulose, but was not affected on other carbon sources, suggesting that *VdST3* was involved in the utilization of galactose, maltose and cellulose.

### Down-regulation of *VdST12* by asiRNAs decreases the pathogenicity of *V. dahliae*

**Figure 7:**
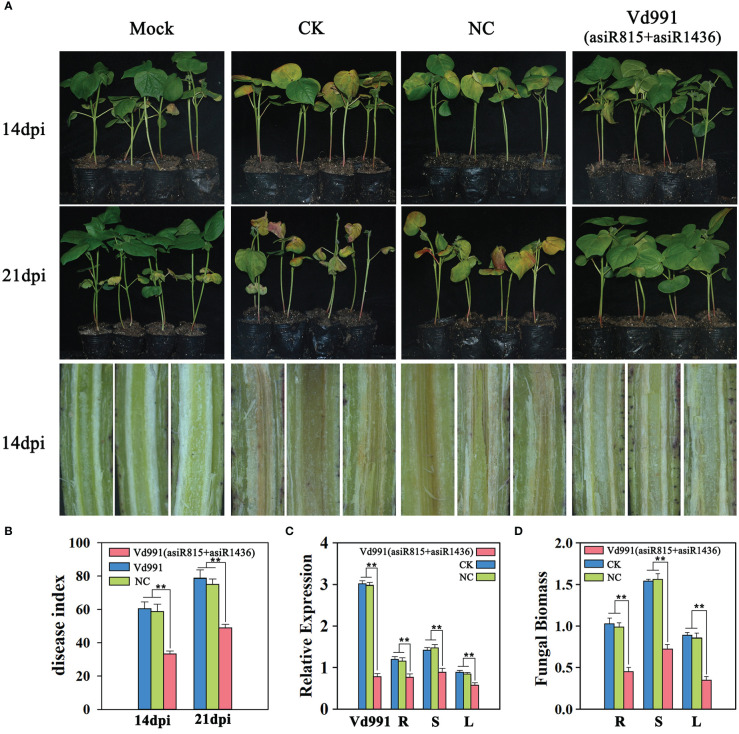
Effect of asiRNAs (asiRNA815+asiRNA1436) targeting *VdST12* on the pathogenicity of *V. dahliae*. **(A)** Fungal infection symptoms of cotton seedlings infected by Vd991 (asiRNA815+asiRNA1436). Wild-type Vd991 was used as control (CK), and Vd991 co-cultured with nematode asiRNA was used as negative control (NC). **(B)** Disease index of seedlings infected by Vd991 (asiRNA815+asiRNA1436) at 14 dpi and 21 dpi. **(C)** The expression level of *VdST12* in Vd991 (asiRNA815+asiRNA1436) was detected at 6 days after co-culture and seedlings infected by Vd991 (asiRNA815+asiRNA1436) was detected at 14 dpi by qRT-PCR analysis. **(D)**. qRT-PCR measurement of fungal biomass in seedlings infected by Vd991 (asiRNA815+asiRNA1436) at 21 dpi. The data were statistically analyzed by the IBM SPSS statistics 26.0. Statistical significance was determined using Student’s t-test. Asterisks (**) above the error bars indicated significant differences at p < 0.01 between seedlings infected by asiRNA-treated Vd991 and wild-type Vd991 (CK) and NC.

The asiRNAs (asiR815 or asiR1436) targeting *VdST12* could effectively inhibit the growth of *V. dahliae*, Vd991 co-cultured with asiR815 and asiR1436 were therefore used for the following infection process assay. Cotton seedlings (Xinluzao 7) at two-leaf stage were inoculated with wild-type Vd991 (CK), Vd991 co-cultured with nematode asiRNA (NC), or Vd991 co-cultured with asiR815+asiR1436. At 14 and 21 dpi, seedlings inoculated with Vd991 (asiR815+asiR1436) showed milder symptoms compared with the seedlings inoculated with CK and NC (**Figure 7A**).

The disease indexes of cotton seedlings infected with Vd991 (asiR815+asiR1436) at 14 dpi and 21 dpi (DI=33.3 and 48.9, respectively) were significantly lower than that of CK (DI=60.5 and 78) and NC (DI=58.9 and 75.9) at the corresponding time point (**Figure 7B**). The stem dissection experiments showed that the browning of seedlings inoculated with Vd991 (asiR815+asiR1436) was significantly lighter than that of CK and NC (**Figure 7A**). qRT-PCR was used to determine the relative expression level of *VdST12* in Vd991 (asiR815+asiR1436) and seedlings inoculated with Vd991 (asiR815+asiR1436). It was found that the expression level of *VdST12* in Vd991 (asiR815+asiR1436) was reduced significantly compared to CK and NC. In different tissues (root, stem and leaf) of cotton seedlings infected with Vd991 (asiR815+asiR1436), a significant reduction of *VdST12* was also observed compared to the same tissue of the CK and NC cotton seedlings. These results suggest that asiRNAs (asiR815+asiR1436) successfully suppressed the expression of *VdST12* in Vd991 and such suppression could be maintained in the subsequent fungal growth in cotton plants, including roots, stems and leaves (**Figure 7C**). Quantification of fungal biomass showed that seedlings infected with Vd991 (asiR815+asiR1436) accumulated less fungal biomass compared to the CK and NC seedlings(**Figure 7D**). Taken together, asiRNAs targeting *VdST12* could decrease the pathogenicity of *V. dahliae*, suggesting the involvement of *VdST12* in the pathogenic process of *V. dahliae*.

## Discussion

In this study, we identified a total of 65 sugar transporter genes in *V. dahliae*, and analyzed their gene structure and protein motifs. It was found that most of VdST proteins possessed 10-12 TMDs, and 8 proteins harbored only 7-9 TMDs, likely due to sequence deletion during gene evolution. Consistent with this, similar ST protein structures have been observed in many plants and other fungi (Afoufa-Bastien et al., 2010; Reuscher et al., 2014; Lv et al., 2020). A phylogenetic analysis found that the 65 *VdST* genes were grouped into 8 Clades with specificity to different groups of sugar molecules, which was similar to research in other fungi (Peng et al., 2018; Lv et al., 2020). Compared with Clade I, VI and VII, Clade IV, II, V, VIII and III harbored more members, mainly including hexose transporters, disaccharide (lactose and maltose) permeases, myoinositol transporters and quinate permeases. Most of the VdST proteins were predicted to be plasma membrane-localized transporters and are capable of acquisition of monosaccharide and disaccharide substrates, including glucose, galactose (VDAG_01215, VDAG_08381 and VDAG_05443), xylose (VDAG_03925), alpha-glucoside, lactose and maltose. It was notable that several genes encoding inositol transporters and quinine permeases were identified from *V. dahliae*. The function of these genes in fungus has hardly been reported, and further studies on their roles in growth and pathogenicity of *V. dahliae* are needed.

*V. dahliae* invades cotton through the root system, therefore, the biological effect of the root exudates is expected to be crucial for successful infection of *V. dahliae*. It was found that the content of carbohydrate and the amount of amino acids in the root exudates of susceptible variety were distinctly more than those of resistant ones (Yuan et al., 2002). Root exudates from the susceptible cotton varieties but not from the resistant cotton varieties promoted the growth of *V. dahliae* (Yuan et al., 2002; Wu et al., 2007). *V. dahliae* responded to all kinds of root exudates but more strongly to those from susceptible variety than to those from tolerant and resistant varieties (Zhang et al., 2020). The genes whose expression level was significantly up-regulated after induction in root exudates from susceptible varieties were considered to be related to pathogenicity of *V. dahliae* (Xu et al., 2018; Zhang et al., 2020). To find the *ST* genes important for pathogenicity of *V. dahliae*, the expression profiles of all 65 *VdST* genes in response to root exudates from susceptible and resistant varieties were investigated based on the RNA-seq datasets we generated previously (Zhang et al., 2020), and 9 of them were found to be induced by root exudates from susceptible cotton variety. Sugar can act not only as an energy storage material, but also as a signal molecule. Sugar can regulate the expression of sugar transporter gene for sugar metabolism (Ozcan and Johnston, 1999; Horák, 2013; Kim et al., 2013). Therefore, the high sugar content in root exudates from susceptible cotton variety may be responsible for the high expression level of the 9 sugar transporter genes, which can be used as candidate genes for further functional study.

With the completion of genome sequencing of *V. dahliae* and application of genomics, transcriptomics and proteomics information, a number of genes important for growth, infection and pathogenicity of *V. dahliae* have been identified (Gao et al., 2010; Tzima et al., 2012; Liu et al., 2013a; Tian et al., 2014; Fan et al., 2017; Zhang et al., 2017; Luo et al., 2019). At present, a powerful mean to elucidate the function of *V. dahliae* genes is to obtain knock-out mutants *via* homologous recombination, which is a mature technology and has been used in many studies (Rauyaree et al., 2005; Tzima et al., 2011; Liu et al., 2013b; Saitoh et al., 2014). However, it often increases the actual workload due to its low efficiency and sometimes gets undesirable results (Takahashi et al., 2006; Krappmann, 2007). In this study, artificial small interfering RNAs (asiRNA) were used to verify the function of *V. dahliae* genes for the first time. It was found that feeding asiRNAs targeting *VdST3* and *VdST12* could decrease their expression level in *V. dahliae*, resulting in reduction of the fungal hyphae and colony diameter and decrease of pathogenicity. The results obtained by asiRNA assay have been found to be consistent with that achieved by loss-of function experiments in corresponding fungal genes (Guo et al., 2011; Guo et al., 2019). Therefore, asiRNA assay could serve as a quick prescreening to identify genes important for growth and pathogenicity of *V. dahliae*.

In addition, asiRNAs targeting important genes can be used as exogenous reagents to enhance plant disease resistance, providing ideas for further using asiRNA technology to control the occurrence of Verticillium wilt. The growth of fungus was severely inhibited by foliar spray of asiRNAs or dsRNAs targeting genes related to pathogenicity of pathogens (Koch et al., 2016; Wang et al., 2016; Guo et al., 2019; Qiao et al., 2021). *V. dahliae* is a root pathogen. RNA is unstable in soil so direct application of dsRNA or asiRNA to the soil cannot protect the plant from *V. dahliae* infection. Although it has been reported that pretreatment of roots with dsRNAs targeting genes related to *V. dahliae* virulence inhibited the infection of *V. dahliae* (Qiao et al., 2021), this is not a practical solution for commercial cotton production, therefore, studies are required to find strategies for stabilizing RNA in soil. Compared with dsRNAs, the asiRNAs used in this study are easier to be synthesized with a lower cost.

Sugar transporter genes of fungi participate in the interaction between host plants and fungi and play an important role in the absorption of host sugars (Doidy et al., 2012). The hexose transporter protein UfHXT1 in *Bacillus subtilis* is specifically expressed in the haustorium and directly located on the haustorium membrane. UfHXT1 can transport glucose and fructose, which are hydrolysates derived from host sucrose (Voegele and Mendgen, 2003; Voegele and Mendgen, 2011). UmSRT1 of *Ustilago maydis* has a high affinity for sucrose, it competes with the host ZmSUT1 for sucrose (Carpaneto et al., 2005; Wahl et al., 2010). The preference of sugar uptake and utilization by sugar transporters of *V.dahliae* has not been reported yet. Sugar transporters of *V. dahliae* were found to be associated with different sugars in this study. *VdST12* was found to be involved in the utilization of xylose, galactose, maltose and cellulose, and *VdST3* to be involved in the utilization of galactose, maltose and cellulose.

Among many plant disease control methods, breeding disease resistant varieties is an important control measure that is harmless to people and animals and friendly to the environment. Growing evidence has indicated that RNAi technology can be used in crop protection (Zotti et al., 2018). HIGS, an RNAi-based technology, has been a favorable tool for obtaining disease resistant plants and identifying important gene functions (Zhang et al., 2016; Qi et al., 2018; Guo et al., 2019; Sang and Kim, 2020). HIGS strategy has been successfully used to suppress *V. dahliae* infection and improve disease resistance of plants (Wang et al., 2016; Zhang et al., 2016; Song and Thomma, 2018; Xu et al., 2018). HIGS can be performed by either generating stable transgenic plants or using transient expression systems mainly based on recombinant viral vector systems. In this study, transient HIGS in cotton plants silenced *VdST3* and *VdST12* transcripts of *V. dahliae* in host plants and enhanced cotton resistance to Verticillium wilt, indicating that these genes are potential target candidates for generation of stable disease resistant varieties *via* HIGS in the future.

## Conclusions

*VdST3* and *VdST12* are two sugar transporter genes required for growth and pathogenicity of *V. dahliae*. The findings of this study demonstrated that TRV-based HIGS in cotton plants silenced *VdST3* or *VdST12* transcripts of *V. dahliae* in the hosts, leading to inhibition of fungal biomass and enhancement of cotton’s resistance against *V. dahliae*. The asiRNAs targeting *VdST12* and *VdST3* could transiently silence *VdST12* and *VdST3*, leading to suppression of growth and pathogenicity of *V. dahliae*, with a more significant suppression observed for asiRNAs targeting *VdST12*. Our results provided candidate target genes and alternative solutions for enhancing cotton disease resistance.

## Data availability statement

The datasets presented in this study can be found in online repositories. The names of the repository/repositories and accession number(s) can be found in the article/**Supplementary Material**.

## Author contributions

Conceptualization, LC, YL, and JS. Investigation, LC, BC, and TS. Writing-original draft preparation, LC, XZ, and FL. Writing-review and editing, LC, YL, and QZ. Supervision, YL and JS. Project administration, XZ and JS. All authors contributed to the article and approved the submitted version.
