# Supplementary material for: Identification of sugar transporter genes and their roles in the pathogenicity of Verticillium dahliae on cotton
Source: Front Plant Sci. 2023 Jan 26;14:1123523. doi: 10.3389/fpls.2023.1123523 (PMC9910176; doi:10.3389/fpls.2023.1123523)
Supplement: Supplementary file 1 [file Image_1.pdf]

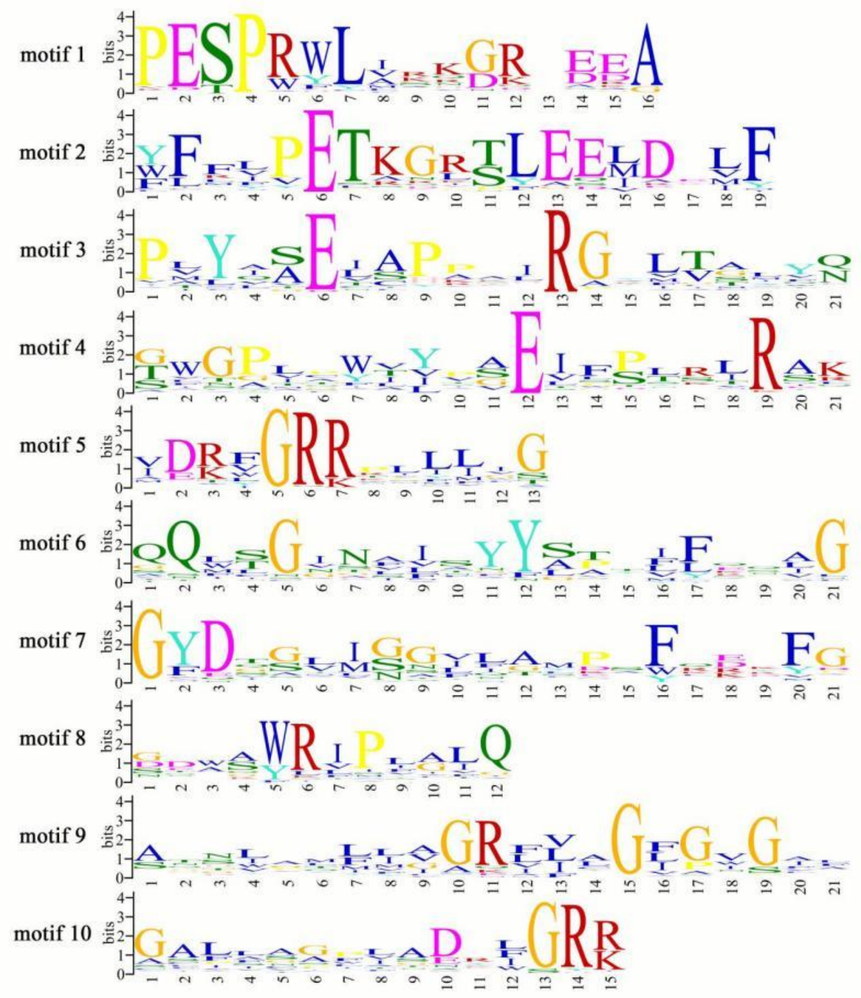

Supplementary Figure S1. Sequence logos for 10 motifs of VdST domains with the MEME program. MEME motifs were displayed by stacks of letters at each site. The total height of the stack is the 'information content' of that site in the motif in bits. The height of each letter in a stack is the probability of the letter at that site multiplied by the total information content of the stack. The x-axis indicated the width of the motif, and y-axis represented the bits of each letter.

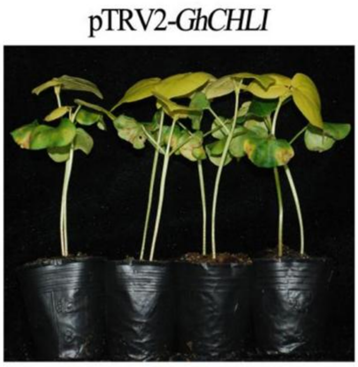

Supplementary Figure S2. Leaf-bleaching phenotype of seedlings treated with pTRV2-*GhCHLI* at 10 days after injection.
